# Plasma and cerebrospinal fluid inflammation and the blood-brain barrier in older surgical patients: the Role of Inflammation after Surgery for Elders (RISE) study

**DOI:** 10.1186/s12974-021-02145-8

**Published:** 2021-04-30

**Authors:** Sarinnapha M. Vasunilashorn, Long H. Ngo, Simon T. Dillon, Tamara G. Fong, Becky C. Carlyle, Pia Kivisäkk, Bianca A. Trombetta, Kamen V. Vlassakov, Lisa J. Kunze, Steven E. Arnold, Zhongcong Xie, Sharon K. Inouye, Towia A. Libermann, Edward R. Marcantonio, RISE Study Group, Sharon K. Inouye, Steven Arnold, Bradford Dickerson, Tamara Fong, Richard Jones, Towia Libermann, Edward R. Marcantonio, Thomas Travison, Simon T. Dillon, Jacob Hooker, Tammy Hshieh, Long Ngo, Hasan Otu, Annie Racine, Eva M. Schmitt, Alexandra Touroutoglou, Sarinnapha Vasunilashorn, Ayesha Abdeen, Douglas Ayres, Brandon Earp, Jeffrey Lange, Gregory Brick, Antonia Chen, Robert Davis, Jacob Drew, Richard Iorio, Fulton Kornack, Michael Weaver, Anthony Webber, Richard Wilk, Lisa Kunze, David Shaff, Kamen Vlassakov, Brett Armstrong, Angelee Banda, Sylvie Bertrand, Madeline D’Aquila, Jacqueline Gallagher, Baileigh Hightower, Shannon Malloy, Jacqueline Nee, Chloe Nobuhara, Abigail Overstreet, Bianca Trombetta, David Urick, Guoquan Xu, Grae Arabasz, Michael Brickhouse, Regan Butterfield, Shirley Hsu, Sara Makaretz, Judit Sore, Fan Chen, Yun Gou, Douglas Tommet, Sabrina Carretie, Ted Gruen, Katherine Tasker

**Affiliations:** 1 Department of Medicine, Beth Israel Deaconess Medical Center, Boston, MA USA; 2 Harvard Medical School, Boston, MA USA; 3 Department of Epidemiology, Harvard T. H. Chan School of Public Health, Boston, MA USA; 4 Department of Biostatistics, Harvard T. H. Chan School of Public Health, Boston, MA USA; 5 Department of Neurology, Beth Israel Deaconess Medical Center, Boston, MA USA; 6 Marcus Institute for Aging Research, Boston, MA USA; 7 Department of Neurology, Massachusetts General Hospital, Boston, MA USA; 8 Department of Anesthesia, Brigham and Women’s Hospital, Boston, MA USA; 9 Department of Anesthesia, Beth Israel Deaconess Medical Center, Boston, MA USA; 10 Department of Anesthesia, Massachusetts General Hospital, Boston, MA USA

**Keywords:** Inflammation, Neuroinflammation, Plasma, Cerebrospinal fluid, Blood-brain barrier

## Abstract

**Background:**

Our understanding of the relationship between plasma and cerebrospinal fluid (CSF) remains limited, which poses an obstacle to the identification of blood-based markers of neuroinflammatory disorders. To better understand the relationship between peripheral and central nervous system (CNS) markers of inflammation before and after surgery, we aimed to examine whether surgery compromises the blood-brain barrier (BBB), evaluate postoperative changes in inflammatory markers, and assess the correlations between plasma and CSF levels of inflammation.

**Methods:**

We examined the Role of Inflammation after Surgery for Elders (RISE) study of adults aged ≥ 65 who underwent elective hip or knee surgery under spinal anesthesia who had plasma and CSF samples collected at baseline and postoperative 1 month (PO1MO) (*n* = 29). Plasma and CSF levels of three inflammatory markers previously identified as increasing after surgery were measured using enzyme-linked immunosorbent assay: interleukin-6 (IL-6), C-reactive protein (CRP), and chitinase 3-like protein (also known as YKL-40). The integrity of the BBB was computed as the ratio of CSF/plasma albumin levels (Qalb). Mean Qalb and levels of inflammation were compared between baseline and PO1MO. Spearman correlation coefficients were used to determine the correlation between biofluids.

**Results:**

Mean Qalb did not change between baseline and PO1MO. Mean plasma and CSF levels of CRP and plasma levels of YKL-40 and IL-6 were higher on PO1MO relative to baseline, with a disproportionally higher increase in CRP CSF levels relative to plasma levels (CRP tripled in CSF vs. increased 10% in plasma). Significant plasma-CSF correlations for CRP (baseline *r* = 0.70 and PO1MO *r* = 0.89, *p* < .01 for both) and IL-6 (PO1MO *r* = 0.48, *p* < .01) were observed, with higher correlations on PO1MO compared with baseline.

**Conclusions:**

In this elective surgical sample of older adults, BBB integrity was similar between baseline and PO1MO, plasma-CSF correlations were observed for CRP and IL-6, plasma levels of all three markers (CRP, IL-6, and YKL-40) increased from PREOP to PO1MO, and CSF levels of only CRP increased between the two time points. Our identification of potential promising plasma markers of inflammation in the CNS may facilitate the early identification of patients at greatest risk for neuroinflammation and its associated adverse cognitive outcomes.

## Background

Insights into the relationship of plasma and cerebrospinal fluid (CSF) biomarkers may help to advance our pathophysiologic understanding of how peripheral events like surgery might trigger neuroinflammation. Our understanding of the inter-relationship between plasma and CSF remains incomplete, and as a consequence, our ability to identify blood-based biomarkers of the surgical effect, including inflammation and neuroinflammation, remains limited. A proposed model highlights the potential associated downstream consequences of surgery: individuals predisposed to a heightened inflammatory response when exposed to an acute stressor, such as surgery or infection, are at increased risk for longer-term adverse outcomes [1–7]. Under certain conditions, these systemic inflammatory mediators are hypothesized to activate brain microglia, leading to neuroinflammation, which if sustained, may cause permanent neuronal injury (as illustrated in [1]).

From this posited neuroinflammatory hypothesis, two prominent questions remain. First, how does an acute stressor such as surgery impact the BBB after a prolonged period of time? Second, can we identify blood-based markers indicative of neuroinflammation? Such questions require understanding the complex nature of molecular dynamics underscoring the protein levels in peripheral blood and the central nervous system (CNS), which can be influenced by many factors.

To better understand the complex relationship between peripheral and CNS markers of inflammation before and after surgery, we evaluated three key knowledge gaps in a study of older adults undergoing major elective surgery under spinal anesthesia. Our specific aims were (1) to examine whether surgery compromises the blood-brain barrier (BBB, measured from CSF-plasma albumin ratio [Qalb]) 1 month post-surgery; (2) to evaluate whether changes in levels of inflammatory markers following surgery are greater in plasma and CSF at 1 month post-operation (PO1MO) relative to preoperation (PREOP or baseline); and (3) to examine 3 previously identified plasma markers of inflammation associated with surgery for their correlations with CSF levels: interleukin (IL)-6, C-reactive protein (CRP), and chitinase-3-like protein 1 (CHI3L1, also known as YKL-40). We hypothesized that (1) surgery would compromise the BBB, resulting in higher permeability of proteins observed 1 month following surgery; (2a) mean PO1MO levels of all three inflammatory markers would be higher on PO1MO compared to PREOP; (2b) CSF levels would exhibit disproportionately higher increases in these inflammatory markers following surgery compared with plasma; (3a) plasma markers of inflammation would have high correlations with CSF; and (3b) higher plasma-CSF correlations would be observed on PO1MO compared with PREOP.

## Methods

### Study sample

The Role of Inflammation after Surgery in Elders (RISE) study is a cohort study aimed to assess the correlation of blood plasma, CSF, and imaging biomarkers of inflammation in patients aged 65 years or older who underwent elective hip or knee arthroplasty under spinal anesthesia. The overall study design and protocol have been described [8]. Briefly, patients were enrolled if they had planned admission for at least 24 h and surgery scheduled at least 15 days in advance to allow time for preoperative testing. Exclusion criteria have been previously described and include safety exclusions for lumbar puncture and magnetic resonance imaging.

The Institutional Review Board of Partners Healthcare System (Massachusetts General Hospital, Brigham and Women’s Hospital, Brigham and Women’s Faulkner Hospital) approved all study procedures, with ceded review from Beth Israel Deaconess Medical Center and Hebrew SeniorLife, the study coordinating center.

### Specimen collection

Phlebotomy was performed on patients at three time points: baseline (at home or during preadmission testing clinic visit), postoperative day 1 (POD1), and approximately 1 month postoperatively (PO1MO). During processing, plasma and cellular material were separated using low-speed centrifugation (1500 relative centrifugal force [rcf]), subaliquoted, and stored at − 80C.

CSF was acquired preoperatively during induction of spinal anesthesia (baseline) and at PO1MO via a research lumbar puncture. CSF was collected via dropwise collection or aspiration directly into collection tubes. To minimize potential blood contamination of the CSF, samples were centrifuged at 1000 rcf for 10 min prior to storage at − 80^o^C in low absorption polypropylene tubes. This paper focuses on the baseline and PO1MO time points since CSF was only available at these two time points.

### Immunoassays

Plasma and CSF levels of three inflammatory markers (IL-6, CRP, and YKL-40) and of albumin were measured using sandwich assays: the fully automated Ella System SinglePlex cartridge (ProteinSimple San Jose, CA; kit part # SPCKB-PS-000200) for the inflammatory markers, and an enzyme-linked immunosorbent assay from Abcam (Cambridge, MA; ab108788) run on a semiautomatic Tecan Freedom Evo liquid handling platform (Männedorf, Switzerland) for albumin. The limit of detection was 1.64 pg/ml for CRP, 0.26 pg/ml for IL-6, and 3.74 pg/ml for YKL-40. Qalb was defined as CSF albumin/plasma albumin × 10^−3^. Coefficient of variations (CVs) of duplicate measures were generally < 5%. If a CV was > 10%, the assay was repeated.

### Statistical analysis

To determine correlation within biofluids (i.e., plasma-plasma or CSF-CSF) and between biofluids (i.e., plasma-CSF), we examined Spearman correlation coefficients. All analyses were conducted using SAS 9.4 (SAS Institute, Cary, NC).

## Results

Table 1 reports the characteristics of our study sample, presenting means and standard deviations, as well as proportions, for the sample of patients with complete plasma and CSF biospecimen data at both baseline and PO1MO (*n* = 29). Patients were on average age 75 and mostly female, 10% had ≥ 2 Charlson comorbidities, and all patients underwent spinal anesthesia alone. Fifty-two percent of patients underwent total knee arthroplasty and 48% underwent total hip arthroplasty. The average hospital length of stay was 3.2 days.

**Table 1:** Sample characteristics of the RISE study patients with complete biospecimen data (plasma and cerebrospinal fluid) at both baseline and postoperative 1 month

	Complete biospecimen data for BL and PO1MO (*N* = 29)
Age, M ± SD	74.8 ± 4.6
Female, *n* (%)	20 (69%)
Non-white, *n* (%)	2 (7%)
Charlson comorbidity index, *n* (%)	
0	24 (83%)
1	2 (7%)
2+	3 (10%)
Anesthesia type	
Spinal alone, *n* (%)	29 (100%)
Surgery type, *n* (%)	
Total knee arthroplasty	15 (52%)
Total hip arthroplasty	14 (48%)
Hospital length of stay, M ± SD	3.2 ± 0.8

*Abbreviations*: *BL* baseline, *M* mean, *PO1MO* postoperative 1 month, *SD* standard deviation

**Fig. 1:**
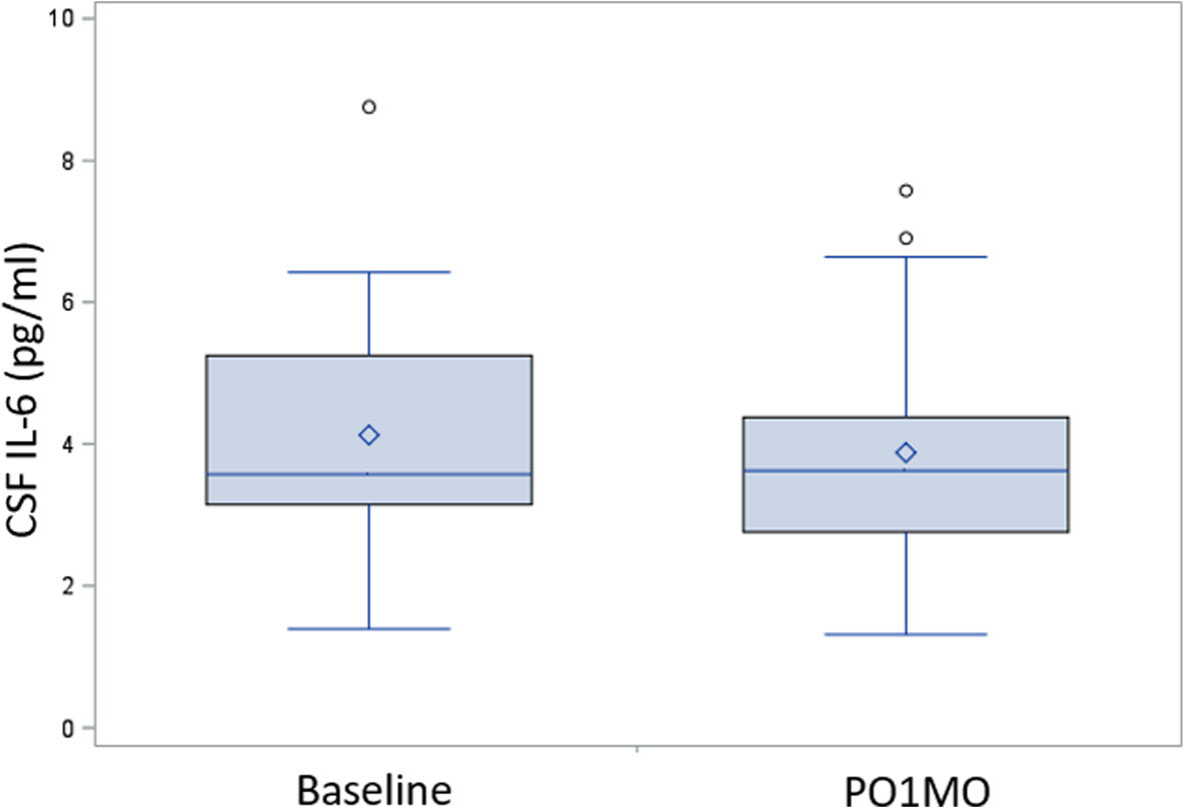
Box and whisker plots of interleukin-6 (IL-6) cerebrospinal fluid (CSF) levels at baseline and postoperative 1 month* (PO1MO). *Highest value for PO1MO (130.0 pg/ml) not plotted to facilitate visualization of the distributions. The height of the box represents the interquartile range (the distance between the 25th and 75th percentiles). The horizontal line in the box interior represents the group median. The vertical lines (whiskers) extending from the box indicate the group minimum value and maximum value within the “upper fence” (i.e., 1.5*IQR + Quartile 3). The diamond symbol represents the group mean, and the circle symbols represent outlying values that extend beyond the upper fence

Table 2 reports the distributions of albumin, IL-6, CRP, and YKL-40 in the sample with complete plasma and CSF biospecimen data (*n* = 29). Between baseline and PO1MO, we observed three general patterns. First, mean Qalb was similar between the baseline and PO1MO time points (6.42 for both); a small number of patients had Qalb ≥ 9.0 (4 patients [14%] at baseline and 2 patients (7%) on PO1MO, including the one patient having Qalb ≥ 9.0 at both time points), indicating compromised BBB [3]. Second, IL-6 levels increased for plasma and CRP levels increased for both plasma and CSF. Average plasma IL-6 levels increased from 3.7 pg/ml (baseline) to 5.4 pg/ml (PO1MO), and average CSF IL-6 was similar between baseline and PO1MO 4.1 pg/ml and 3.9 pg/ml* (respectively; *with 1 outlier > 130 pg/ml removed; see Fig. 1 for box-whisker plot). Increases in CRP levels were observed between the two time points: average plasma CRP levels increased from 7.4 mg/l (baseline) to 8.2 mg/l (PO1MO), and average CSF CRP tripled between baseline (0.01 mg/l) and PO1MO (0.03 mg/l). Third, YKL-40 increased in plasma only (96.8 ng/ml [baseline] to 135.7 ng/ml [PO1MO]), while CSF levels remained near equivalent at baseline (280.1 ng/ml) and PO1MO (280.0 ng/ml).

**Table 2:** Distribution of markers within the analytic sample (complete bispecimen data for both baseline and PO1MO, *N* = 29)

		Data available at both BL and PO1MO			
Time period	Biofluid	Mean ± SD	Median	Min	Max
Albumin (g/l)					
Baseline	CSF	0.18 ± 0.07	0.17	0.08	0.34
	Plasma	28.64 ± 7.33	27.10	16.52	52.84
	CSF/plasma ratio (× 10^−3^)	6.42 ± 2.62	6.64	2.31	14.20
PO1MO	CSF	0.17 ± 0.06	0.17	0.07	0.34
	Plasma	28.06 ± 7.48	27.25	18.97	57.11
	CSF/plasma ratio (× 10^−3^)	6.42 ± 2.76	6.17	1.98	15.22
Interleukin-6 (pg/ml)					
Baseline	Plasma	3.67 ± 1.68	3.21	1.52	7.33
	CSF	4.05 ± 1.58	3.49	1.31	8.67
PO1MO	Plasma	5.41 ± 3.37	4.37	2.23	15.90
	CSF*	3.91 ± 1.56	3.65	1.34	7.60
C-reactive protein (mg/l)					
Baseline	Plasma	7.41 ± 13.21	2.52	0.56	56.12
	CSF	0.01 ± 0.01	0.01	0.00	0.07
PO1MO	Plasma	8.18 ± 10.45	4.77	1.24	53.34
	CSF	0.03 ± 0.04	0.01	0.00	0.17
Chitinase 3-like protein (ng/ml)					
Baseline	Plasma	96.76 ± 107.44	60.11	23.01	573.21
	CSF	280.08 ± 62.46	286.47	131.11	378.19
PO1MO	Plasma	135.68 ± 122.95	96.06	30.14	524.60
	CSF	279.96 ± 61.30	292.23	142.02	396.58

*Abbreviations*: *CSF* cerebrospinal fluid, *PO1MO* postoperative 1 month, *SD* standard deviation

*Values for the outlier (> 130 pg/ml) removed (*n* = 28)

Table 3 reports the Spearman correlation coefficients of plasma and CSF inflammatory markers at baseline (*n* = 29). Between biofluids (plasma and CSF; shown in red), three significant correlations were identified — all including CSF levels of CRP with plasma levels at baseline of itself (CRP: *r* = 0.70, *p* < .01), IL-6 (*r* = 0.58, *p* < .01), and YKL-40 (*r* = 0.43, *p* < .05).

**Table 3:**
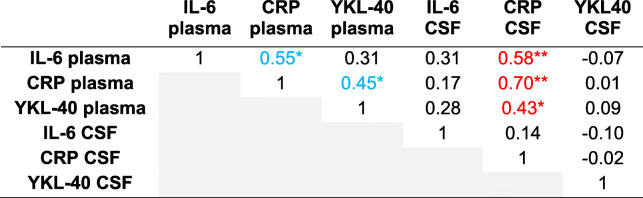
Spearman correlation coefficients of biofluids measured at baseline (complete biospecimen data for both baseline and PO1MO; *n* = 29)

*p < .05, **p < .01

*Abbreviations*: *CRP* C-reactive protein, *CSF* cerebrospinal fluid, *IL-6* interleukin-6, *YKL-40* chitinase 3-like protein 1 (CHI3L1/YKL-40)

Blue indicates significance (*p* < .05) within biofluid correlation (i.e., plasma-plasma or CSF-CSF), and red indicates significance between biofluid correlation (i.e., plasma-CSF)

Table 4 lists the Spearman correlation coefficients of plasma and CSF inflammatory markers at PO1MO (*n* = 29). Similar to baseline, IL-6, YKL-40, and CRP plasma levels were significantly correlated with CRP CSF levels, all assessed at 1 month (*r* = 0.47, 0.59, and 0.89, respectively). Additionally, IL-6 plasma and IL-6 CSF levels were moderately correlated at P01MO (*r* = 0.48, *p* < .01). When we conducted the same analyses using the full dataset of RISE study patients with blood and CSF at either the baseline (*n* = 57) or the PO1MO (*n* = 40) time point, the overall findings remained similar (Tables 5 and 6, respectively).

**Table 4:**
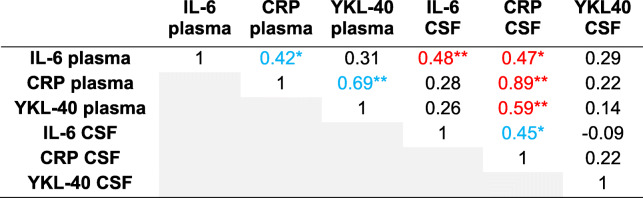
Spearman correlation coefficients of biofluids measured on postoperative 1 month (complete biospecimen data for both baseline and postoperative 1 month; *n* = 29)

*p < .05, **p < .01

*Abbreviations*: *CRP* C-reactive protein, *CSF* cerebrospinal fluid, *IL-6* interleukin-6, *YKL-40* chitinase 3-like protein 1 (CHI3L1/YKL-40)

Blue indicates significance (*p* < .05) within biofluid correlation (i.e., plasma-plasma or CSF-CSF), and red indicates significance between biofluid correlation (i.e., plasma-CSF)

**Table 5:**
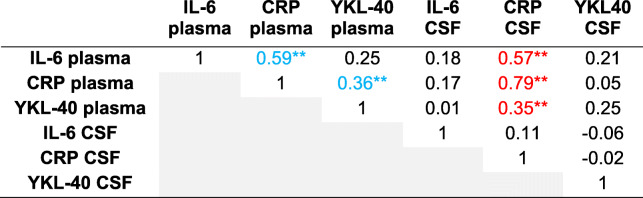
Spearman correlation coefficients of biofluids measured at baseline (all available data at baseline; *n* = 57)

*p < .05, **p < .01

*Abbreviations*: *CRP* C-reactive protein, *CSF* cerebrospinal fluid, *IL-6* interleukin-6, *YKL-40* chitinase 3-like protein 1 (CHI3L1/YKL-40)

Blue indicates significance (*p* < .05) within biofluid correlation (i.e., plasma-plasma or CSF-CSF), and red indicates significance between biofluid correlation (i.e., plasma-CSF)

**Table 6:**
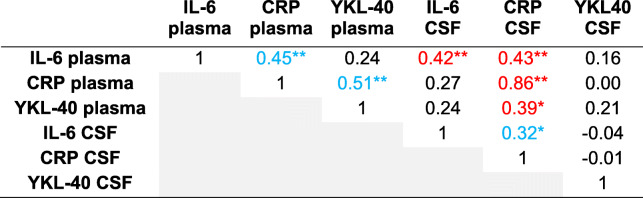
Spearman correlation coefficients of biofluids measured on postoperative 1 month (all available data at baseline; *n* = 40)

*p < .05, **p < .01

*Abbreviations*: *CRP* C-reactive protein, *CSF* cerebrospinal fluid, *IL-6* interleukin-6, *YKL-40* chitinase 3-like protein 1 (CHI3L1/YKL-40)

Blue indicates significance (*p* < .05) within biofluid correlation (i.e., plasma-plasma or CSF-CSF), and red indicates significance between biofluid correlation (i.e., plasma-CSF)

We additionally examined the Spearman correlation coefficients of plasma and CSF inflammatory markers between baseline and PO1MO. Within both plasma and CSF, every protein was significantly correlated with itself between the two time points. Spearman *r*’s for plasma IL-6, CRP, and YKL-40 measured on baseline and PO1MO were 0.49, 0.52, and 0.80 (*p* < .01 for all), respectively; Spearman *r*’s for CSF IL-6, CRP, and YKL-40 measured on baseline and PO1MO were 0.50, 0.58, and 0.93 (*p* < .01 for all), respectively. The only significant correlation between biofluids at the two time points was observed between baseline levels of YKL-40 plasma and PO1MO CSF levels of CRP (*r* = 0.51, *p* < .01).

Baseline Qalb (our measure of BBB integrity) was correlated with inflammatory markers at both baseline and PO1MO. Qalb at baseline was correlated with (1) baseline CRP CSF levels (*r* = 0.48, *p* < .01) and (2) PO1MO levels of plasma CRP, CSF CRP, and CSF IL-6 (*r* = 0.45, 0.47, and 0.38 [respectively], *p* < .05 for all). There was good correlation between Qalb baseline and Qalb PO1MO (*r* = 0.64, *p* < .01).

## Discussion

**Fig. 2:**
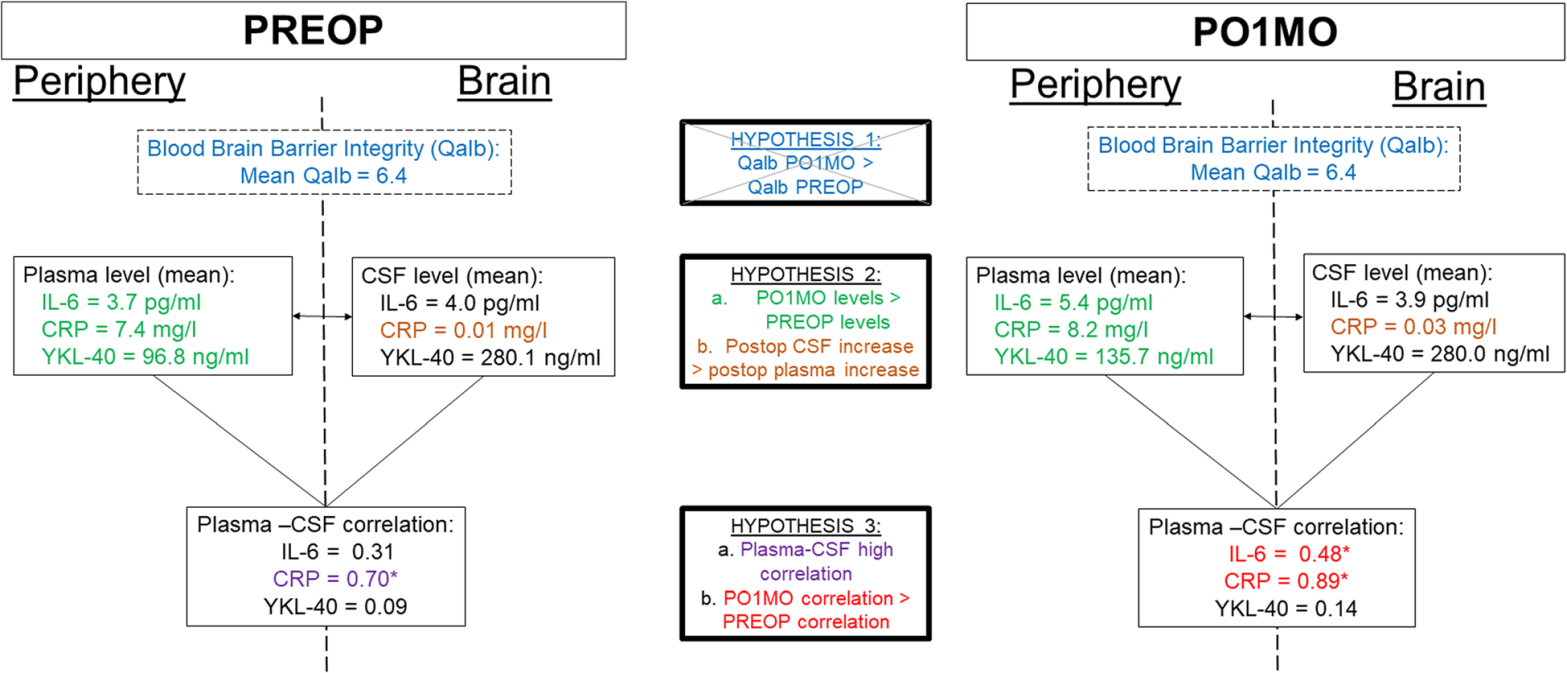
Summary of findings related to the posited hypotheses. Abbreviations: CSF cerebrospinal fluid, IL-6 interleukin-6, Qalb CSF albumin-plasma albumin ratio, PREOP preoperative, PO1MO postoperative 1 month, YKL-40 chitinase 3-like protein 1. *p < .01. Each hypothesis and its associated empirical result are shown in blue for hypothesis 1, green for hypothesis 2a, orange for hypothesis 2b, purple for hypothesis 3a, and red for hypothesis 3b

In this study of older adults undergoing major noncardiac surgery, we found evidence in support of two of our three hypotheses (summarized in Fig. 2). Our first hypothesis was not supported; we did not observe compromises in the integrity of the BBB between PREOP and PO1MO based on the CSF-albumin ratio (hypothesis 1). For our second hypothesis, we found that mean plasma and CSF levels of CRP and plasma levels of YKL-40 and IL-6 were higher on PO1MO relative to PREOP levels (hypothesis 2a). Additionally, there was a disproportionately higher increase in CRP CSF levels relative to plasma levels between PREOP and PO1MO (tripled in CSF vs. 10% increase in plasma) (hypothesis 2b). For our third hypothesis, we observed significant plasma-CSF correlations for CRP (PREOP and PO1MO) and IL-6 (PO1MO only) (hypothesis 3a), with higher correlations on PO1MO compared to PREOP (hypothesis 3b).

At both baseline and PO1MO, we found that plasma and CSF levels of CRP were well correlated and that levels of plasma IL-6 and levels of plasma YKL-40 were correlated with CSF CRP. At PO1MO only, plasma and CSF levels of IL-6 were correlated. Despite these between biofluid correlations, we were ultimately unable to identify promising blood-based inflammatory markers of CNS inflammation, based on our knowledge of the origins of these inflammatory markers. As an example, since CRP is considered to be primarily produced in the periphery (predominantly in the liver [9, 10]), it is more probable that our findings reflect a “leaking” of CRP from the periphery (i.e., plasma) into the CNS (i.e., CSF) than “leakage” from the CNS into the periphery. Interestingly, the increased levels of CSF inflammatory proteins at PO1MO occurred despite no change in Qalb, suggesting that BBB integrity for other proteins may not be fully reflected in this measure. Given this, it is unlikely that plasma CRP, IL-6, or YKL-40 are promising blood-based markers of CSF inflammation. We also found that (1) peripheral markers of inflammation correlated well with other peripheral inflammatory markers within the same time point and (2) for plasma and CSF, each inflammatory marker was correlated with itself between the baseline and PO1MO time points.

Although not observed in our study, compromises in BBB have been associated with surgery in humans and animal models [11–17]. In mice, anesthesia and/or surgery may induce age-associated BBB permeability, as determined by immunohistochemistry imaging and spectrophotometric quantification [18]. Among patients undergoing cardiac surgery, postsurgical disruption of the BBB detected using magnetic resonance imaging (MRI) was observed in 47% of the 19 patients, all of whom had no clinical evidence of a stroke or delirium at the time of gadolinium administration or the MRI scan [19]. The absence of changes in BBB permeability between baseline and PO1MO in our healthy sample of older RISE patients may be due to the fact that post-surgery disruptions in the BBB may have resolved by the 1-month post-surgery time point or may be related to the poor sensitivity of Qalb to detect changes in BBB permeability since Qalb does not fully capture BBB dysfunction, as specific changes in vessels and BBB properties may not be reflected in Qalb [13, 20]. Ideally, we would have examined additional measures of BBB integrity or Qalb closer to the surgical event (e.g., postoperative day 1 [POD1]); however, we were limited in the measures we could assay and collection of CSF at the POD1 time point was not part of the RISE protocol.

Plasma and CSF correlations of inflammatory markers seem to be dependent on the characteristics of the study sample. Among 141 patients with Alzheimer’s disease (AD), a good correlation between plasma and CSF levels of IL-6 levels was reported (*r* = 0.76, *p* < .001) [21]. In contrast, no correlation between IL-6 plasma and CSF levels was observed in 173 older adults who were asymptomatic for AD (*r* = 0.16, *p* = .05) [22]. Our findings in older surgical adults (none of whom had known pre-existing dementia) yielded a plasma-CSF correlation of IL-6 levels that was between the Sun et al. [21] and Bettcher et al. [22] publications (*r* = 0.48, *p* < .01 on PO1MO). This suggests that, for IL-6, the relationship between plasma and CSF may be influenced by factors associated with cognition (e.g., BBB integrity or presence of AD), although we were unable to adequately probe this possibility within our small sample. Although Qalb did not increase between baseline and PO1MO in our sample, the plasma-CSF IL-6 correlations suggest the possibility of (1) increased permeability for small proteins, such as IL-6 and CRP (IL-6 is approximately 20 kDa and CRP 23 kDa in size versus the size of albumin [about 60 kDa] [23–26]); (2) IL-6 may be stimulating CNS IL-6 production indirectly (irrespective of the integrity of the BBB) [27]; or (3) stimuli that induce IL-6 expression in peripheral blood mononuclear cells also induce the expression in microglial cells since plasma and CSF levels of IL-6 were observed to be relatively similar at baseline.

Among the inflammatory markers examined, we found preliminary evidence for the possibility of stronger plasma-CSF correlations at the PO1MO time point. For instance, CRP exhibited medium to high correlations at baseline (*r* = 0.70, *p* < .01) and extremely high correlation at PO1MO (*r* = 0.89, *p* < .01). These promising findings highlight the importance of further examining these relationships in larger surgical cohorts*.* It also underscores the value of considering other modalities for measuring neuroinflammation, including [^11^C] PBR28 on PET imaging. In the RISE study, we recently reported that between the baseline and PO1MO time points, inflammation measured by [^11^C] PBR28 on PET imaging decreased [28]. Technical issues with PBR28, as described in the manuscript, may explain this surprising result. The findings reported in our current manuscript better align with that of our previous work in a separate, larger cohort of patients undergoing elective surgery [6, 29].

We highlight several study strengths. RISE applied state-of-the art approaches to the collection of biospecimens and detailed clinical data on older patients undergoing major surgery, including the collection of plasma and CSF. Our empirically driven analysis examined potential correlations between plasma and CSF, with a focus on the longer-term effects of surgery (1 month post-operation). This facilitated further probing of correlations and the integrity of the BBB following surgery over a longer time frame than may not have been previously observed in the literature.

Some study limitations warrant mention. First, this is a relatively small study which will need confirmation; yet it provides important descriptive information to better understand BBB in the context of the longer-term effects of surgery and to identify peripheral markers of neuroinflammation. Given the sample size, we did not adjust for multiple testing, control for covariates, or examine alternate means of considering nonlinearities in the relationship between plasma and CSF levels. We ultimately employed a biomarker “correlational” discovery approach to generate hypotheses of peripheral-CNS relationships that will require confirmation in larger cohorts, for which we are currently enrolling and intend to pursue more rigorous statistical analyses in future work. Second, it may be that our limited set of inflammatory markers does not appropriately represent indicators of inflammation in the CNS. For instance, macrophage inflammatory protein (MIP-1β, also known as CCL4), previously observed to be moderately correlated in plasma and CSF among older adults without AD (*r* = 0.55, [22]), may be a promising additional inflammatory marker for future examination. Ultimately, the identification of a blood-based marker of neuroinflammation will require identification of an inflammatory marker that is produced entirely, or nearly entirely in the CNS (and not in the periphery). Third, at baseline, there was more variability in the time interval between blood and plasma acquisition compared to the collection of both biofluids at PO1MO, which was almost always done at the same time. As previously noted, this may explain the generally stronger correlations observed on PO1MO relative to baseline. Fourth, since all patients underwent spinal anesthesia, we cannot rule out possible protective effects where spinal anesthesia may mediate the relationships observed. Fifth, we acknowledge that consideration of plasma-CSF correlations shortly following surgery (i.e., at the postoperative day 1 [POD1] time point) would have been particularly informative to understanding the role of surgery on inflammation and neuroinflammation; however, collection of CSF on POD1 was not part of the RISE protocol. Last, we use CSF levels of inflammation as the “gold standard” for neuroinflammation given the absence of an alternative approach, such as brain tissue in the RISE study. It remains challenging to contextualize baseline CSF levels of these inflammatory markers within the RISE study sample since CSF levels of, for example, CRP, in community-dwelling adults are not currently well-described (though average plasma CRP levels in our study are higher than population-based studies of older adults) [30].

In summary, we found that all three plasma inflammatory markers (CRP, IL-6, and YKL-40) were higher at 1 month post-surgery than at baseline, but of these, only CRP was higher in the CSF. Moreover, there was a greater increase in CRP CSF levels relative to plasma levels between the two time points. In contrast, the integrity of the BBB was similar between the two time points. The plasma-CSF correlation results suggest that CRP plasma-CSF correlations are high at all time points, likely due to leakage of peripheral CRP into the CNS. Future studies in larger surgical populations will facilitate the identification of blood-based markers of neuroinflammation and understanding of CNS inflammatory disorders. Our planned next steps include investigation of the relationships of these inflammatory markers with delirium and postoperative neurocognitive status. The ability to identify markers of neuroinflammation would facilitate the monitoring of changes in the brain via blood-based markers with the ultimate aim of identifying patients at greatest risk for neuroinflammation and its associated adverse cognitive outcomes.

## Data Availability

The datasets analyzed during the current study are available from the corresponding author on reasonable request.
